# Evaluation of Antimicrobial Peptides in Saliva as Potential Therapeutic Agents Against Oral Pathogens in Pakistan

**DOI:** 10.7759/cureus.73758

**Published:** 2024-11-15

**Authors:** Rabia Asad, Muhammad Asif Shahzad, Sana Knawal, Shaher Bano, Mariyah Javed, Ammara Anwar, Syed Shahab Ud Din Shah

**Affiliations:** 1 Department of Community and Preventive Dentistry, Akhtar Saeed Medical and Dental College, Lahore, PAK; 2 Department of Oral and Maxillofacial Surgery, Azra Naheed Dental College, Superior University, Lahore, PAK; 3 Office of the Registrar, University of Medical and Dental College, Faisalabad, PAK; 4 Department of Oral Biology, Rahbar College of Dentistry, Lahore, PAK; 5 Department of Oral Medicine, Rahbar College of Dentistry, Lahore, PAK; 6 Department of Biochemistry, Faculty of Biological Sciences, Quaid-i-Azam University, Islamabad, PAK

**Keywords:** antimicrobial peptides, oral health, oral pathogens, pakistan, salivary peptides, therapeutic agent

## Abstract

Background: Maintaining optimal oral health is essential for overall well-being; however, conditions such as dental caries and gingivitis remain prevalent in Pakistan and are further worsened by increasing antibiotic resistance.

Objective: To evaluate the antimicrobial properties of salivary peptides as potential therapeutic agents against common oral pathogens in Pakistan.

Methodology: A one-year cross-sectional study was conducted in Lahore, Pakistan, at Sharif Medical and Dental College and Akhter Saeed Medical and Dental College, involving 384 participants aged 18-65 years. High-performance liquid chromatography (HPLC) was used to isolate antimicrobial peptides (AMPs) from oral swabs and saliva samples. Using the enzyme-linked immunosorbent test (ELISA), the minimum inhibitory concentration (MIC) of histatin, defensin, and cathelicidin against *Streptococcus mutans* and *Candida albicans* was determined. T-tests and other statistical analyses were used to assess the significance of the results across demographic variables.

Results: There were 210 men (54.69%) and 174 women (45.31%) in the participation group. A total of 298 individuals (77.60%) did not smoke, while 221 participants (57.56%) said they brushed their teeth every day. Moreover, 198 individuals (51.56%) had oral infections with *S. mutans* alone, 71 participants (18.49%) had oral pathogens with *C. albicans* alone, and 52 people (13.54%) had both. Histatin of 8.09 ± 2.13 µg/mL, defensin of 7.83 ± 2.31 µg/mL, and cathelicidin of 6.19 ± 1.57 µg/mL were the MIC values for salivary AMPs against *S. mutans*. MIC values for histatin, defensin, and cathelicidin against *C. albicans* were 10.57 ± 1.82 µg/mL, 9.01 ± 2.03 µg/mL, and 7.42 ± 1.73 µg/mL, respectively. Males had higher MIC values than females, and there were significant variations according to smoking status (p < 0.05) and age (p < 0.05), suggesting that smokers had lower AMP effectiveness.

Conclusion: Salivary AMPs, particularly histatin, defensin, and cathelicidin, demonstrate strong potential as therapeutic alternatives against oral infections such as dental caries and gingivitis, suggesting a promising strategy to mitigate antibiotic resistance in Pakistan. Further research is needed to explore their application in clinical practice.

## Introduction

Quality of life, well-being, and even systemic health are profoundly influenced by oral health, which plays a critical role in overall health [1]. In Pakistan, the prevalence of oral diseases, such as dental caries, periodontitis, and gingivitis, is driven by high levels of bacterial and fungal pathogens in the oral cavity. These conditions are exacerbated by factors such as a lack of dental hygiene education, inadequate public health initiatives, and restricted access to preventive care facilities, especially in rural and underserved areas [2,3]. In particular, vulnerable groups such as low-income populations, smokers, and individuals with chronic illnesses (e.g., diabetes) are disproportionately affected. These pathogens disrupt the oral microbiome's balance, leading to various oral diseases that not only impact oral health but also have the potential to cause systemic infections [4].

The overuse of antibiotics and chemical agents such as chlorhexidine in treating oral infections has become increasingly problematic due to the rapid rise in antibiotic-resistant strains. These therapies are not only losing effectiveness but also contribute to microbial resistance, making it harder to control infections and leading to recurring oral health issues [5]. This situation emphasizes the urgent need for alternative approaches that are effective and less prone to resistance. This problem is especially severe in Pakistan, where the worrying rise of drug-resistant microbial strains is a result of antibiotic abuse. Novel treatment strategies that are both efficient and less likely to develop resistance are thus desperately needed [6,7].

A potential remedy for this problem is antimicrobial peptides (AMPs). In humans and other species, AMPs are naturally occurring chemicals that are a component of the innate immune system [8]. Targeting bacteria, viruses, and fungi, AMPs are well-known for their broad-spectrum antibacterial action. They may also improve wound healing and alter immunological responses [9]. AMPs break down microbial cell membranes in a manner that reduces the possibility of resistance, in contrast to conventional antibiotics [10]. Histatins, defensins, and cathelicidins - AMPs that are naturally found in saliva - act as the body's first line of defense against oral pathogens, preserving a healthy microbiome and preventing infection [11].

In Pakistan, investigating the therapeutic potential of salivary AMPs may result in safer, locally effective substitutes for antibiotics in the treatment of oral infections such as dental caries, periodontitis, and gingivitis, which are primarily caused by *Streptococcus mutans* and *Candida albicans*, particularly for high-risk populations such as the elderly and those with impaired immune systems. This discovery may open the door for AMP-based formulations designed to address the region's particular microbial ecosystem and healthcare concerns by concentrating on the particular antibacterial qualities of these peptides.

Research objective

This study aimed to evaluate the antimicrobial properties of peptides present in human saliva to assess their potential as therapeutic agents against common oral pathogens in Pakistan.

## Materials and methods

Study design and setting

The cross-sectional design study was carried out at the Sharif Medical and Dental College Lahore and Akhter Saeed Medical and Dental College Lahore, Pakistan, employing its excellent laboratory capabilities for sample collection and analysis. The research ran over one year, from March 2023 to April 2024, to ensure a full examination of the AMPs.

Inclusion and exclusion criteria

Participants were selected using a random stratified convenience sampling technique to enhance representation across various demographic strata. Eligible participants were adults between the ages of 18 and 65 years who provided informed consent and had not taken antibiotics in the preceding three months. The age range was chosen to include a broad demographic relevant to oral health while excluding those too young to consent. Exclusion criteria included individuals with severe systemic illnesses, known salivary gland disorders, or treatments affecting saliva production.

Sample size

The World Health Organization (WHO) algorithm, which predicts the number of participants required for adequate statistical analysis, was used to determine the sample size for this investigation. The computation produced a necessary sample size of around 384 individuals with a 95% confidence level (Z = 1.96), an estimated prevalence of oral infections set at 50% (p = 0.5), and a 5% margin of error (e = 0.05). This sample size guarantees sufficient statistical power to assess salivary peptides' antibacterial capabilities against oral infections.

WHO Sample Size Formula

The formula for calculating sample size is the following:

n = Z^2^ × p (1- p)/e^2^

Where:

n = required sample size

Z = 1.96 (for 95% confidence level)

p = estimated prevalence (as a decimal)

e = margin of error (as a decimal)

Sample Size Calculation

Let's assume:

Confidence level: 95% (thus, 𝑍=1.96)

Estimated prevalence of oral pathogens: 𝑝= 0.5 (50% prevalence is commonly used for maximum sample size estimation)

Margin of error: 𝑒 = 0.05 (5%)

Substituting the Values:

Calculate Z2: = (1.96)^2^ ≈ 3.8416

Calculate p (1- p): p × (1- p) = 0.5 × 0.5 = 0.25

Calculate e^2^: = (0.5)^2^ = 0.0025

Substituting into the formula:

n = 3.8416 × 0.25/0.0025 = 384.16

After rounding, the required sample size (n) is approximately 384 participants.

Data collection

Participants' saliva and oral swab samples were collected under standardized conditions in a specialized clinical sampling space. In order to collect saliva samples, participants were instructed to pool their saliva at the bottom of their mouth and gently eject it into sterile, polypropylene collecting tubes using a non-stimulatory passive drooling approach. Because of their suitability for cold storage, which guarantees less peptide degradation, these tubes were selected. Participants were advised to abstain from eating, drinking, smoking, and any oral hygiene activities for at least an hour before the collection in order to improve uniformity and prevent contamination. In order to account for diurnal fluctuations in salivary composition, which may have an effect on peptide concentration, samples were always taken early in the morning.

To maintain the stability of the AMP throughout transportation, the saliva tubes were immediately put on ice after collection. They were then sent to the laboratory within two hours for timely processing. Sterile Dacron swabs were used to obtain oral swab samples from the tongue and buccal mucosa. The samples were then put in a Cary-Blair transport medium to ensure the survival of any microbial pathogens while being transported to the laboratory. Prior to sample collection, informed permission was obtained from each participant, guaranteeing adherence to ethical guidelines for studies involving human beings. Throughout the research, participants received assurances of the confidentiality and anonymity of their data.

Antimicrobial peptide isolation and quantification

Saliva samples were centrifuged at 4°C as they arrived at the lab in order to eliminate cellular debris. After being meticulously gathered and aliquoted into separate microcentrifuge tubes that were made specially to reduce protein binding, the supernatant was kept at -80°C until analysis. The separation of certain peptides such as histatins, defensins, and cathelicidins was made possible by the use of high-performance liquid chromatography (HPLC) for AMP isolation based on molecular weight and hydrophobicity. Each peptide was quantified using the enzyme-linked immunosorbent assay (ELISA) after isolation, yielding exact concentration values for analysis.

Oral swab collection and pathogen identification

In order to promote the development of the target organisms, the swab samples were streaked over *S. mutans *and *C. albicans*-specific selective agar medium and then incubated under ideal conditions. Colonies were examined morphologically and biochemically after incubation. DNA sequencing was used to confirm microbial species in situations of unclear identification.

Quality control and sample integrity

The findings were subjected to strict quality control methods to guarantee their correctness and repeatability. In compliance with procedures intended to maintain AMP stability, all saliva and swab samples that were not processed right away were kept at -80°C. To ensure AMP quantification uniformity, duplicate samples were made for both HPLC and ELISA. In order to avoid contamination, all handling procedures were carried out in a sterile setting, and equipment calibration was maintained consistently throughout the investigation.

Statistical analysis

Statistical Product and Service Solutions (SPSS, version 26; IBM SPSS Statistics for Windows, Armonk, NY) was used to analyze the data. Microbial prevalence and participant demographics were determined using descriptive statistics. Using techniques such as minimum inhibitory concentration (MIC) determination, the antibacterial activity of salivary peptides was evaluated, and the results were compared across various demographic groups. The significance of the results was assessed using inferential statistics, such as t-tests, with a p-value of less than 0.05 being statistically significant.

## Results

The participant group had an average age of 35.23 years (±10.71). Gender distribution was slightly skewed towards men, with 210 individuals (54.69%) being male and 174 (45.31%) female. Regarding smoking status, 298 participants (77.60%) were non-smokers, while 86 (22.40%) reported smoking. Oral hygiene practices varied, with 221 respondents (57.56%) brushing twice daily or more and 163 (42.44%) brushing less frequently. A history of oral infections was reported by 89 individuals (23.18%). In terms of oral pathogen prevalence, *S. mutans* was found alone in 198 cases (51.56%), *C. albicans *alone in 71 cases (18.49%), both pathogens in 52 cases (13.54%), and no detectable pathogens in 63 cases (16.41%) (Table 1).

**Table 1:** Participant demographics, oral health characteristics, and prevalence of oral pathogens

Characteristic		Descriptive Statistics
Age Range	Mean ± SD	35.23 ± 10.71
Gender (n;%)	Male	210 (54.69%)
	Female	174 (45.31%)
Smoking Status (n;%)	Non-smoker	298 (77.60%)
	Smoker	86 (22.40%)
Oral Hygiene Practices (n;%)	Daily (Twice or more)	221 (57.56%)
	Less than daily	163 (42.44%)
History of Oral Infections	(n;%)	89 (23.18%)
Prevalence of Oral Pathogens (n;%)	Only Streptococcus mutans	198 (51.56%)
	Only Candida albicans	71 (18.49%)
	Both Pathogens	52 (13.54%)
	No Pathogen Detected	63 (16.41%)

The MIC values of three salivary AMPs against *C. albicans* and *S. mutans* are shown in Table 2. Histatin's MIC against *S. mutans* was 8.09 ± 2.13 µg/mL, whereas its MIC against* C. albicans *was 10.57 ± 1.82 µg/mL. Defensin's MIC values against *S. mutans* were 7.83 ± 2.31 µg/mL, whereas its MIC values against *C. albicans* were 9.01 ± 2.03 µg/mL. With MIC values of 6.19 ± 1.57 µg/mL for *S. mutans* and 7.42 ± 1.73 µg/mL for* C. albicans*, cathelicidin demonstrated the lowest levels of effectiveness against these infections.

**Table 2:** Minimum inhibitory concentration (MIC) values of antimicrobial peptides against oral pathogens

Salivary Peptide	Pathogen	MIC (µg/mL) Mean ± SD
Histatin	Streptococcus mutans	8.09 ± 2.13
	Candida albicans	10.57 ± 1.82
Defensin	Streptococcus mutans	7.83 ± 2.31
	Candida albicans	9.01 ± 2.03
Cathelicidin	Streptococcus mutans	6.19 ± 1.57
	Candida albicans	7.42 ± 1.73

The effectiveness of AMPs is compared by demographic parameters in Table 3. For Histatin (8.21 ± 2.02 µg/mL), defensin (7.84 ± 2.13 µg/mL), and cathelicidin (6.21 ± 1.63 µg/mL), men had marginally higher MIC values than females, who had values of 7.93 ± 1.84 µg/mL, 7.42 ± 2.21 µg/mL, and 5.84 ± 1.52 µg/mL, respectively. With histatin at 7.63 ± 1.94 µg/mL, defensin at 7.31 ± 2.32 µg/mL, and cathelicidin at 5.76 ± 1.62 µg/mL, individuals between the ages of 18 and 35 showed lower MICs for all peptides than those between the ages of 36 and 65. Compared to non-smokers (histatin of 7.71 ± 1.73 µg/mL, defensin of 7.42 ± 2.13 µg/mL, cathelicidin of 5.95 ± 1.57 µg/mL), smokers had higher MIC values (histatin of 8.52 ± 2.24 µg/mL, defensin of 8.03 ± 2.36 µg/mL, and cathelicidin of 6.38 ± 1.89 µg/mL), which may indicate a possible decline in AMP efficacy linked to smoking.

**Table 3:** Comparative efficacy of antimicrobial peptides by demographic characteristics

Demographic Factor		Histatin MIC (µg/mL)	Defensin MIC (µg/mL)	Cathelicidin MIC (µg/mL)
Gender	Male	8.21 ± 2.02	7.84 ± 2.13	6.21 ± 1.63
	Female	7.93 ± 1.84	7.42 ± 2.21	5.84 ± 1.52
Age Group	18-35 years	7.63 ± 1.94	7.31 ± 2.32	5.76 ± 1.62
	36-65 years	8.13 ± 1.82	7.95 ± 2.21	6.14 ± 1.53
Smoking Status	Smoker	8.52 ± 2.24	8.03 ± 2.36	6.38 ± 1.89
	Non-Smoker	7.71 ± 1.73	7.42 ± 2.13	5.95 ± 1.57

The statistical significance of AMP activity against oral infections according to demographic variables is shown in Table 4. The mean MIC values for histatin (8.21 ± 2.02 µg/mL for males, 7.93 ± 1.84 µg/mL for females), defensin (7.84 ± 2.13 µg/mL for males, 7.42 ± 2.21 µg/mL for females), and cathelicidin (6.21 ± 1.63 µg/mL for males, 5.84 ± 1.52 µg/mL for females) did not differ statistically significantly. Age-group differences were significant, but younger individuals (18-35 years old) have lower MIC values for histatin (p=0.045), defensin (p=0.037), and cathelicidin (p=0.045). Significant findings were also obtained by smoking status, showing that non-smokers had lower MIC values for histatin (p=0.023), defensin (p=0.012), and cathelicidin (p=0.024), suggesting that smokers had reduced AMP effectiveness.

**Table 4:** Statistical significance of antimicrobial peptide activities against oral pathogens based on demographic factors

Demographic Factor	Peptide	Mean MIC (µg/mL) Male	Mean MIC (µg/mL) Female	p-value
Gender	Histatin	8.21 ± 2.02	7.93 ± 1.84	0.325
	Defensin	7.84 ± 2.13	7.42 ± 2.21	0.278
	Cathelicidin	6.21 ± 1.63	5.84 ± 1.52	0.205
Age Group	Histatin	8.13 ± 1.82	7.63 ± 1.94	0.045
	Defensin	7.95 ± 2.21	7.31 ± 2.32	0.037
	Cathelicidin	6.14 ± 1.53	5.76 ± 1.62	0.045
Smoking Status	Histatin	8.52 ± 2.24	7.71 ± 1.73	0.023
	Defensin	8.03 ± 2.36	7.42 ± 2.13	0.012
	Cathelicidin	6.38 ± 1.89	5.95 ± 1.57	0.024

## Discussion

The results of the research show that salivary AMPs have the potential to be used as therapeutic agents against prevalent oral infections in Pakistan; however, there are notable variations in their effectiveness depending on the demographic group. According to our study's MIC values, cathelicidin had the lowest MIC against *C. albicans *(7.42 ± 1.73 µg/mL) and *S. mutans *(6.19 ± 1.57 µg/mL), indicating a comparatively greater level of antibacterial activity. Histatin and defensin, on the other hand, demonstrated greater MIC values for both infections, with histatin's MIC values against *S. mutans *being 8.09 ± 2.13 µg/mL and against *C. albicans* being 10.57 ± 1.82 µg/mL. Given that cathelicidin is well known for its strong antibacterial qualities in a variety of settings, our results are in line with earlier research that documented comparable effectiveness patterns [12,13].

Significant patterns were also seen when AMP effectiveness was compared by demographic variables, especially between age groups. For all peptides, participants between the ages of 18 and 35 had reduced MIC values; histatin's MIC was 7.63 ± 1.94 µg/mL. On the other hand, MIC values for histatin were higher in older subjects (36-65 years), suggesting a possible decline in AMP effectiveness with age (8.13 ± 1.82 µg/mL). This finding is consistent with other studies that hypothesize age-related alterations in immunological response and saliva composition may impact AMPs' antibacterial capabilities [14]. The necessity for specialized treatment approaches for this population is highlighted by the higher MIC values seen in older persons, which may possibly be a result of a greater incidence of chronic illnesses or weakened immune systems.

Furthermore, in comparison to non-smokers (7.71 ± 1.73 µg/mL), smokers had higher MIC values (8.52 ± 2.24 µg/mL for histatin), indicating that smoking status had a considerable influence on AMP efficacy. This result supports earlier research that showed smoking negatively impacts dental health and salivary antibacterial activity [15]. Tobacco's toxic ingredients may change the makeup of saliva, which will impact AMPs' functionality and capacity to fight infections. Therefore, our results emphasize how crucial it is to take lifestyle variables into account when assessing salivary AMPs' potential for therapeutic use.

Last but not least, the research participants' total oral pathogen prevalence (51.56% for *S. mutans *and 18.49% for *C. albicans*) highlights the critical need for efficient treatment substitutes, especially in communities with limited access to traditional dental care. Since their distinct methods of action reduce the possibility of resistance development, AMPs may provide a viable option given the growing rates of antibiotic resistance [16,17]. This study lays the groundwork for future research into the therapeutic uses of salivary AMPs in the treatment of oral infections and provides insightful information about their effectiveness.

Strength and limitations

This study's primary strength lies in its use of random stratified sampling, which ensures broad representation across various demographic groups, enhancing the reliability and generalizability of the findings. The rigorous methodologies employed, including HPLC and ELISA, ensure precise and accurate quantification of salivary AMPs. These advanced techniques enable the study to assess the antimicrobial potential of AMPs in detail, providing valuable insights into their therapeutic prospects. The study includes important demographic variables such as age, smoking status, and oral hygiene practices, offering a comprehensive analysis of factors that may influence AMP efficacy. While the study's cross-sectional design provides valuable data, it does not allow for causal conclusions. The random stratified sampling and methodological rigor mitigate potential biases, ensuring that the findings are both robust and applicable to a wide population.

## Conclusions

This study highlights the therapeutic potential of salivary AMPs as effective agents against oral infections, particularly emphasizing the superior activity of cathelicidin. The results underscore the influence of demographic factors, such as age and smoking status, on AMP effectiveness, with younger, nonsmoking individuals demonstrating significantly lower MIC values. These findings suggest that AMP-based therapies could serve as a viable alternative to conventional treatments, especially in populations facing rising antibiotic resistance. Given the growing prevalence of oral diseases in Pakistan, further research into the application of salivary AMPs is crucial for improving oral health outcomes and combating microbial resistance.

Future investigations should explore the combined use of AMPs with other treatments, such as probiotics or alternative antimicrobial agents, to enhance their therapeutic efficacy against oral pathogens. Understanding the synergistic effects of AMPs alongside other antimicrobial strategies could offer valuable insights into optimizing treatment regimens. Additionally, research focusing on the stability of AMPs and the potential for resistance development in clinical settings is needed. Expanding studies to diverse populations and examining the role of AMPs in various oral diseases will help refine and optimize their use, paving the way for broader therapeutic applications in oral healthcare.
